# Functional outcome of knee arthrodesis with a monorail external fixator

**DOI:** 10.1007/s11751-016-0247-5

**Published:** 2016-02-20

**Authors:** Alfred Cyril Roy, Sandeep Albert, Mohamad Gouse, Dan Barnabas Inja

**Affiliations:** Department of Orthopedics Unit-1, CMC, Vellore, India

**Keywords:** Knee arthrodesis, External fixator, Tuberculous arthritis, Post-septic sequelae, Post-traumatic sequelae

## Abstract

Several methods for obtaining knee arthrodesis have been described in the literature and world; over, the commonest cause for arthrodesis is a failed arthroplasty. Less commonly, as in this series, post-infective or traumatic causes may also require a knee fusion wherein arthroplasty may not be indicated. We present salient advantages along with the radiological and functional outcome of twenty four patients treated with a single monorail external fixator. All patients went on develop fusion at an average of 5.4 months with an average limb length discrepancy of 3 cm (1.5–6 cm). Improvements in functional outcome as assessed by the lower extremity functional score (LEFS), and the SF-36 was significant (*p* = 0.000). Knee arthrodesis with a single monorail external fixator is a reasonable single-staged salvage option in patients wherein arthroplasty may not be the ideal choice. The outcome, though far from ideal, is definitely positive and predictable.

**Electronic supplementary material:**

The online version of this article (doi:10.1007/s11751-016-0247-5) contains supplementary material, which is available to authorized users.

## Introduction

Knee arthrodesis, performed since 1900, is achieved by various methods. Whilst the earliest recorded knee fusion, performed by Prof. Albert of Vienna, was for a flail knee in poliomyelitis, the most common indication today is a failed knee arthroplasty. In the developing world, knee arthrodesis is often performed for sepsis or for arthritis after tuberculosis or trauma.

**Fig. 1:**
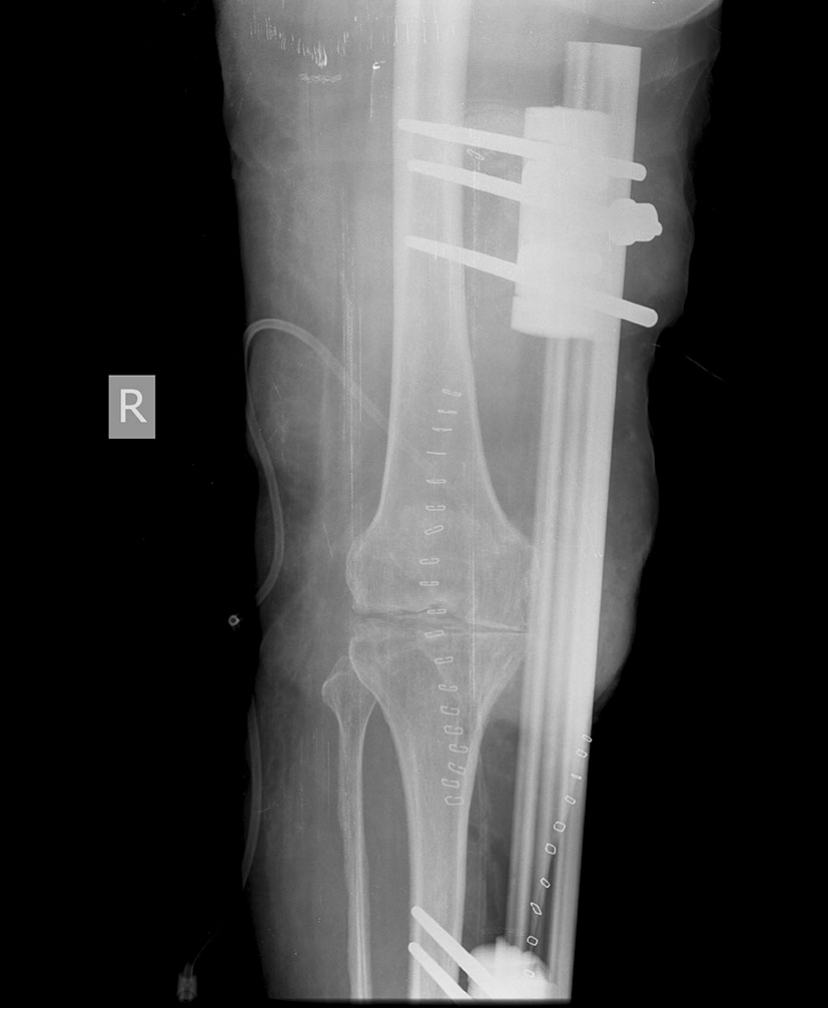
Immediate post-operative antero-posterior plain radiographs showing knee arthrodesis performed with an anterior monorail fixator

**Fig. 2:**
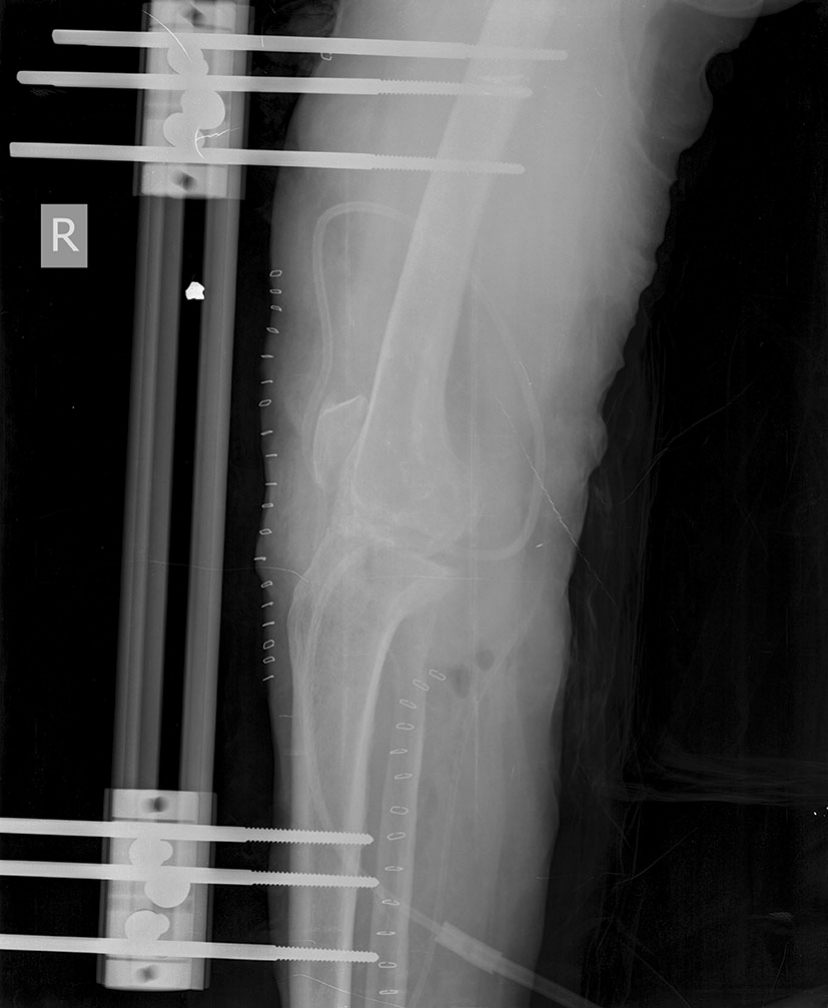
Immediate post-operative lateral plain radiograph showing knee arthrodesis performed with an anterior monorail fixator

Since 1948 [1], external fixators have been utilized to achieve compression across the fusion site. The use of larger diameter radially preloaded half pins has improved fixation and stability. The technique of knee arthrodesis with a monolateral fixator has been described using dynamic axial fixator (DAF, Orthofix SRL, Verona, Italy). The same type of device has been used to bridge bone defects [2]. In this series, we used a monorail external fixator to achieve fixation and compression across the knee for arthrodesis in patients with either post-traumatic or post-septic sequelae (Figs. 1, 2).

## Methodology

Patients who underwent knee arthrodesis for various indications from January 2007 to January 2013 were in included in the study for analysis. Hospital records, clinical photographs, radiographs and follow-up radiographs were analysed, and the functional outcome at final follow-up was recorded. The SF-36 and the LEFS (lower extremity functional score) were utilized for pre-operative and final functional outcome assessment.

### Pre-operative planning and operative technique

Pre-operative planning included a review of the patient’s diagnosis, prior surgical procedures around the knee, the soft tissue condition and the presence of deformities or arthritis in other lower limb large joints. Radiographs were analysed to assess deformity, bone defects and for planning resection margins. The target alignment in arthrodesis of the knee was neutral rotation, flexion of 10–15 degrees and valgus of 7–10 degrees. The patient was positioned supine. A radiolucent table and a tourniquet were used in all the cases. The knee joint was approached through an anterior incision which was modified depending on prior scars. The patella was reflected laterally and either resected or its articular surface removed for the main body to be used as graft to augment fusion. The joint was debrided thoroughly with near total excision of all granulation and granulomatous tissue, inflamed synovium and eburnated cartilage. The distal femur and proximal tibia were then cut appropriately till bleeding surfaces of cancellous bone were encountered. The tibia was cut with a mild posterior and lateral slope to allow for flexion and valgus alignment.

**Fig. 3:**
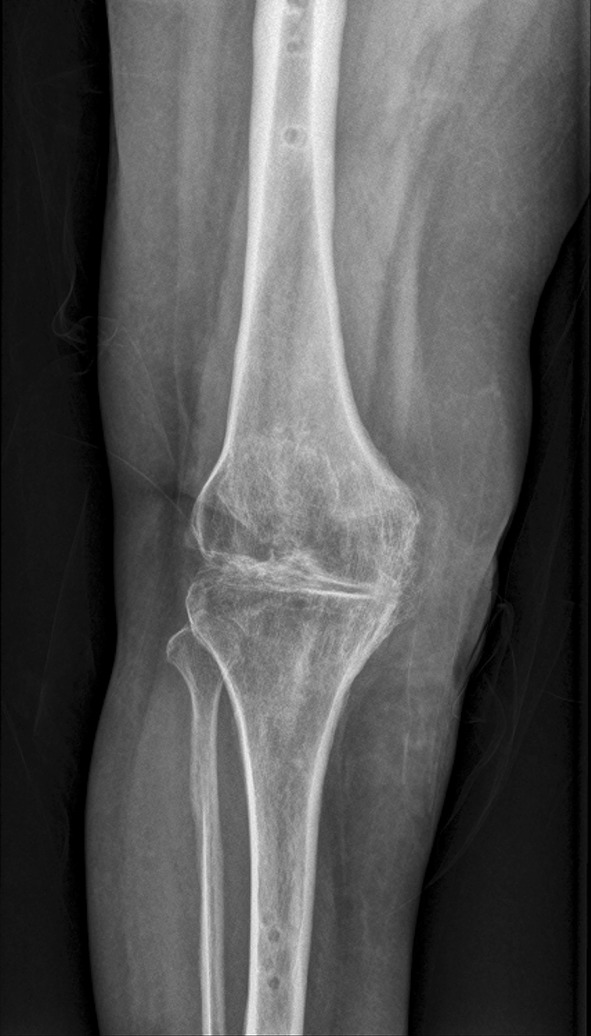
Antero-posterior plain radiograph at seven months with consolidation at the arthrodesis site

**Fig. 4:**
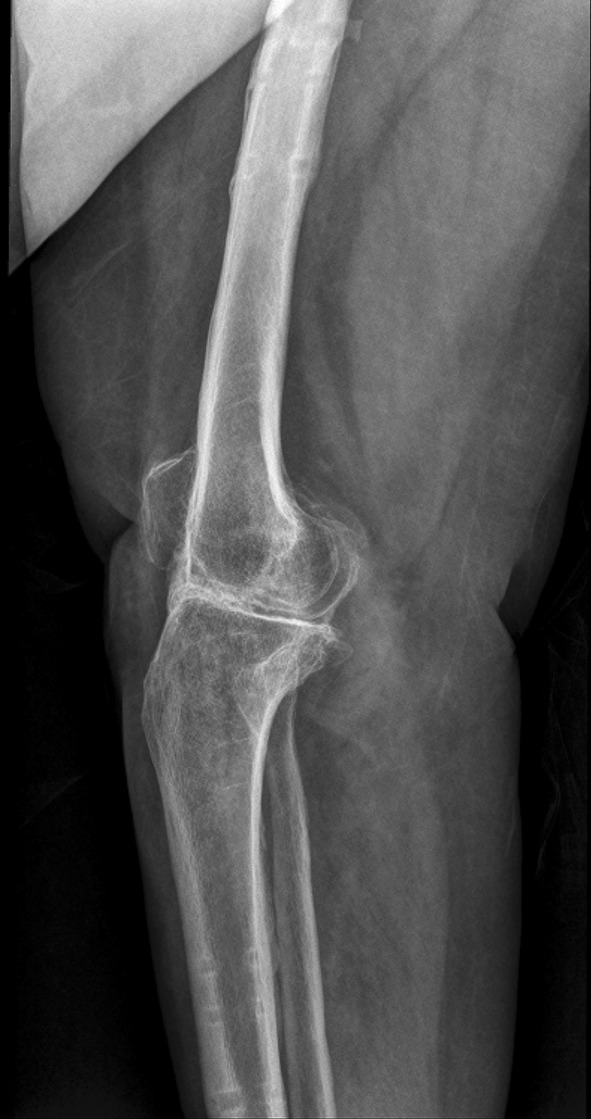
Lateral plain radiograph at seven months showing good consolidation at the arthrodesis site

When the Orthofix monorail was employed anteriorly, the most proximal pin was inserted in the distal diaphysis of the femur ensuring central placement in the sagittal plane. The cut surfaces were then opposed to coapt bleeding surfaces of cancellous bone. The most distal pin on the tibial diaphysis was inserted along the sagittal plane just medial to the tibial crest. This ensured a slight valgus alignment and allowed for central and bicortical placement of the intervening pins. Such placement of the pins ensured positioning of a straight monorail across a slightly valgus knee. All pins (with a minimum of three on either side) were affixed to a single clamp. This facilitated compression when a compression device was utilized in the monorail. When the undersurface of the patella was prepared, the patella was allowed to fall back over the site of fusion with wound closure. Fixation with a screw or pin was not routinely required. Excision of the patella was done in a few cases to simplify closure in a scarred limb with poor soft tissue conditions. The wound was lavaged prior to closure under drains. An adequate distance between the rail and the skin allowed for wound closure and post-operative wound care. This was crucial as with the knee in flexion the monorail fixator was brought closer to the skin anteriorly. At the same time care was taken to avoid having the rail too far from the soft tissue thereby reducing biomechanical stability. There were occasions when additional soft tissue procedures were performed; medial or lateral gastrocnemius flaps served as work horses for defects surrounding the knee. Patients were encouraged to bear weight as tolerated. Partial weight bearing with support was continued for at least 3 months. Radiographs were obtained every 6 weeks for the first 3–6 months. The patients were educated and taught pin site care. Bridging trabeculae and sclerosis with blurring of the cut edges at the fusion site were signs of adequate fusion at which time the patient was usually able to ambulate full weight bearing without support. Frame removal was not routinely preceded by dynamization (Figs. 3, 4).

## Results

**Fig. 5:**
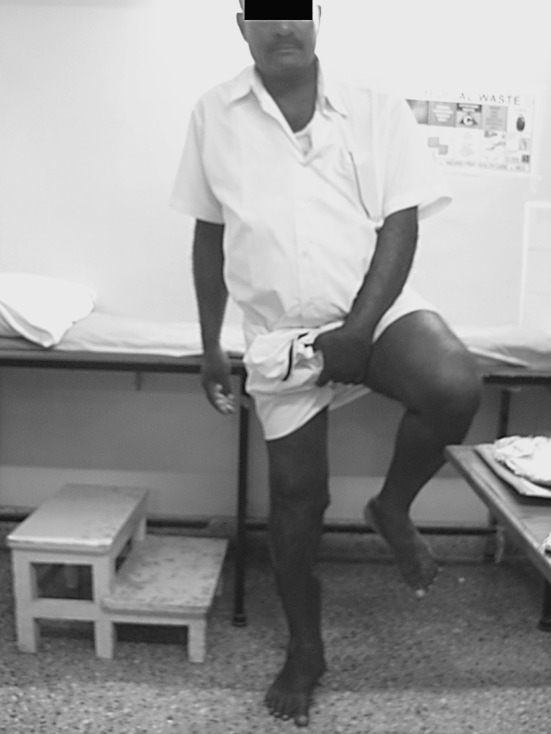
Functional outcome at four years post-knee arthrodesis with an anterior monorail fixator

This study was approved by the institutional review board (IRB no – 8538). The patient demographic data are listed in Table 1. The majority of patients were labourers from agrarian communities. The mean age was 42 years (19–68 years). Articular tuberculosis and other infective sequelae were the majority of causes. Nineteen of the 24 patients had undergone more than one prior surgical procedure including joint debridement, synovectomy or prior fixation for complex trauma. All patients described chronic debilitating pain and deformity affecting function at presentation. Six of 24 patients had wound complications which were managed with a medial or lateral gastrocnemius flap. All 24 patients went on to sound union with no additional intervention. The time to union was 5.4 months on average (4–7 months). None required an additional procedure to augment union. Fourteen of 24 patients were able to return to their pre-injury occupation after frame removal. The average limb length discrepancy was 3 cm (1.5–6 cm). This discrepancy was significantly higher in patients presenting with post-traumatic sequelae as compared to post-infective (5.5 cm vs. 2 cm). The average final valgus alignment was 7 degrees (2–11 degrees.) Nearly all patients had pin site issues especially in the proximal pins which settled with pin site care and oral antibiotics and a few had local antibiotic injections. None of the patients required exchange or revision of pins. The mean pre-operative and post-procedure LEFS and SF scores are given in Table 2. Both the LEFS and SF-36 scores showed significant improvement in the time of frame removal (*p* = 0.000; Fig. 5).

**Table 1:** Patient demographic data

S. no	Age/sex	Host type	Diagnosis	No. of previous surgery index
1	20/M	A	Post-trauma	0
2	40/F	A	Pyogenic	1
3	45/M	A	Pyogenic	2
4	29/M	A	Tuberculosis	2
5	41/M	A	Post-trauma	2
6	68/M	B	Pyogenic	0
7	24/M	A	Post-trauma	2
8	58/M	A	Post-trauma	1
9	33/M	A	Tuberculosis	2
10	48/M	B	Pyogenic	2
11	43/M	A	Tuberculosis	0
12	56/M	A	Post-trauma	2
13	22/M	A	Tuberculosis	0
14	46/M	B	Pyogenic	2
15	69/F	B	Pyogenic	2
16	50/M	A	Tuberculosis	1
17	34/M	B	Tuberculosis	0
18	50/M	B	Tuberculosis	1
19	26/M	A	Tuberculosis	1
20	60/M	B	Pyogenic	1
21	51/M	A	Pyogenic	1
22	28/M	A	Post-trauma	1
23	57/M	A	Post-trauma	2
24	19/M	A	Post-trauma	1

**Table 2:** Pre- and post-scores

Variable	Pre-op	Post-op	*p* value
LEFS	39	64	0.000
SF			
Mental	32	51	0.000
Physical	33	43	0.000

## Discussion

A failed arthroplasty is the most common indication for knee arthrodesis. However, post-traumatic and post-infective sequelae, including tuberculosis, are still encountered in developing countries. In the current era where knee arthroplasty is the dominant treatment for end-stage knee pathologies, combined with heightened patients’ expectations and awareness, arthrodesis as a salvage procedure is often overlooked or even frowned upon. However, arthrodesis may be preferable for certain patients; this would include the patient’s age, occupation and the presence of localized infection. For the younger patient, fusion is a good alternative to staged reconstructive procedures and may be cost effective giving the patient an early return to occupation. In this series, the mean age was 42 years (19–68) with most patients involved in agricultural or hard manual labour where a total knee replacement was not the preferred solution.

The presence of constant disabling pain with deformity and infection for many of these patients had a direct bearing on function as shown by the low pre-operative LEFS and SF-36 scores. A joint debridement along with the stability achieved with compression through the monorail device, coupled with appropriate antimicrobial/antituberculous therapy, provides a conductive environment for fusion. Decrease in pain and improved stability after fusion resulted in an improvement in functional outcome.

Various studies have described the use of external fixators [1, 3, 9, 10], cannulated screws [4], intramedullary devices and other internal fixation (with and without bone graft) [5] for knee fusion. A majority of patients included in these studies included patients who underwent knee fusion following failed or infected knee arthroplasty. The outcomes have varied from 86 to 96 % [6–8] fusion success. Corona et al. and Eralp et al. [9, 10] have also used monolateral external fixation in patients with failed total knee arthroplasty and had union success in 81–100 %. In this series, all patients achieved sound union at final follow-up and most of them returned to their previous occupation. The monorail fixator had the added advantage of patient comfort when compared with bulkier ring external fixators and obviated the need for plaster application as required with the Charnley’s compression device.

Comparison of these data with other study groups was not possible due to patients in this study having had a failed arthroplasty and the average age lower. The outcome parameters in other studies were restricted to assessing limb alignment, length discrepancy and time to fusion. We have further quantified outcome with pre- and post-fusion scores.

Despite the disadvantages of an external fixator, the anterior rail had certain distinct advantages: it performs like a tension band when the limb is loaded; and the ease of application and improved patient comfort in the fixation period. All patients went on to stable fusion which was reflected on their improved scores and clinical outcomes.

## Conclusion

A single anterior rail is a reliable method of obtaining knee fusion for post-infective and post-traumatic sequelae. The technique described offers a single-stage salvage procedure and enables an earlier return to work and occupation. It is a viable alternative over staged reconstructive procedures or complex arthroplasty for certain individuals.

## Electronic supplementary material

Below is the link to the electronic supplementary material.

Supplementary material 1 (AVI 17144 kb)

## References

[1] Charnley J, Lowe HG (1958). A study of the end-results of compression arthrodesis of the knee. J Bone Joint Surg Br.

[2] Conway J (2003) Arthrodesis of the knee using biplanar external fixation. Read at the Annual Meeting of the Limb Lengthening and Reconstruction Society

[3] Brooker AF, Hansen NM (1981). The biplane frame: modified compression arthrodesis of the knee. Clin Orthop.

[4] Lim HC, Bae JH, Hur CR, Oh JK, Han SH (2009). Arthrodesis of the knee using cannulated screws. J Bone Joint Surg Br.

[5] Stiehl JB, Hanel DP (1993). Knee arthrodesis using combined intramedullary rod and plate fixation. Clin Orthop.

[6] Lonner JH, Hershman S, Mont M, Lotke PA (2000). Total knee arthroplasty in patients 40 years of age and younger with osteoarthritis. Clin Orthop.

[7] Gill GS, Chan KC, Mills DM (1997). 5- to 18-year follow-up study of cemented total knee arthroplasty for patients 55 years old or younger. J Arthroplasty.

[8] Mont MA, Lee CW, Sheldon M, Lennon WC, Hungerford DS (2002). Total knee arthroplasty in patients </=50 years old. J Arthroplasty.

[9] Corona PS, Hernandez A, Reverte-Vinaixa MM, Amat C, Flores X (2013). Outcome after knee arthrodesis for failed septic total knee replacement using a monolateral external fixator. J Orthop Surg.

[10] Eralp L, Kocaoğlu M, Tuncay I, Bilen FE, Samir SE (2008). Knee arthrodesis using a unilateral external fixator for the treatment of infectious sequelae. Acta Orthop Traumatol Turc.

